# Cost-effectiveness of first-line erlotinib in patients with advanced non-small-cell lung cancer unsuitable for chemotherapy

**DOI:** 10.1136/bmjopen-2014-006733

**Published:** 2015-07-02

**Authors:** Iftekhar Khan, Stephen Morris, Allan Hackshaw, Siow-Ming Lee

**Affiliations:** 1 CRUK & UCL Cancer Trial Centre, University College London, London, UK; 2 Department of Applied Health Research, University College London, London, UK; 3 University College London Hospital/UCL Cancer Institute, London, UK

**Keywords:** HEALTH ECONOMICS, EPIDEMIOLOGY, STATISTICS & RESEARCH METHODS

## Abstract

**Objective:**

To assess the cost-effectiveness of erlotinib versus supportive care (placebo) overall and within a predefined rash subgroup in elderly patients with advanced non-small-cell lung cancer who are unfit for chemotherapy and receive only active supportive care due to their poor performance status or presence of comorbidities.

**Setting:**

Between 2005 and 2009, a total of 670 patients with non-small cell lung cancer (NSCLC) were randomised across 78 hospital sites (centres) in the UK.

**Participants:**

670 patients with pathologically confirmed stage IIIb-IV NSCLC, unfit for chemotherapy, predominantly poor performance status (>2 on Eastern Cooperative Oncology Group, ECOG) and estimated life expectancy of at least 8 weeks. Patients were followed until disease progression or death, including a subgroup of patients who developed first cycle rash.

**Interventions:**

Patients were randomised (1:1) to receive best supportive care plus oral placebo or erlotinib (150 mg/day) until disease progression, toxicity or death.

**Primary outcome:**

Overall survival (OS).

**Secondary outcomes:**

Progression-free survival (PFS), tumour response and quality adjusted life years (QALY), including within prespecified subgroups.

**Results:**

The mean incremental cost per QALY in all patients was £202 571/QALY. The probability of cost-effectiveness of erlotinib in all patients was <10% at thresholds up to £100 000. However, within the rash subgroup, the incremental cost/QALY was £56 770/QALY with a probability of cost-effectiveness of about 80% for cost-effectiveness thresholds between £50 000 to £60 000.

**Conclusions:**

Erlotinib has about 80% chance of being cost-effective at thresholds between £50 000–£60 000 in a subset of elderly poor performance patients with NSCLC unfit for chemotherapy who develop first cycle (28 days) rash. Erlotinib is potentially cost-effective for this population, for which few treatment options apart from best supportive care are available.

**Trial registration number:**

(ISCRTN): 77383050.

**Strengths and limitations of this study**

- A randomised controlled trial designed prospectively to assess the cost-effectiveness of erlotinib in a predefined subgroup. Rash is used as a retrospective surrogate marker to select patients to continue erlotinib treatment after 4 weeks trial of treatment.
- Quality of Life Data were obtained prospectively up to and beyond progression. In most trials Quality of Life is collected only up to progression.
- More than 99% of patients died, therefore, the uncertainty in modelling future health benefits is largely absent in this analysis.
- This is an important contribution in an area where there is limited data on the cost-effectiveness of cancer treatments in elderly patients.
- The potential economic impact of erlotinib use in the 60% of patients who develop rash is likely to be significant for the UK National Health Service (NHS).

## Introduction

Lung cancer, the leading cause of cancer-related death, accounts for nearly 1.4 million deaths worldwide annually, with a yearly incidence of over 41 000 in the UK,^1^ ^2^ of which 80% are non-small cell lung cancer (NSCLC).^3^ In the USA, the mean monthly cost of treating patients with lung cancer was estimated at £1669 (no active treatment, about £1669; £1=$1.61 US) and £5814 (chemoradiotherapy).^4^ In the UK, the total annual cost of treating lung cancer in 2012 equated to about £2.4 billion^5^—the yearly average cost per patient was £9071. This compares with £2756 for bowel cancer, £1584 for prostate cancer and £1076 for breast cancer. The costs associated with lung cancer are therefore a significant economic burden on healthcare systems worldwide.

It is estimated about 30% of patients with advanced NSCLC do not receive any cytotoxic treatment^6^ because of poor performance status and/or multiple medical comorbidities. The National Institute for Health and Care Excellence (NICE) does not recommend erlotinib treatment for epidermal growth factor receptor (EGFR) mutation-negative patients.^7^ NICE previously recommended erlotinib as an alternative treatment to docetaxel for patients with good performance status who have tried at least one chemotherapy treatment.^8^ The current cost-effectiveness thresholds (willingness to pay) proposed by NICE are £20 000–£30 000 per QALY; however, for special cases, such as end-of-life treatments, supplementary guidance was issued, which suggested a higher threshold to £50 000/QALY.

The recently published TOPICAL trial^9^ was a randomised (1:1), double blind, phase III multicentre trial conducted in the UK comparing erlotinib (ER) with placebo in predominantly elderly patients receiving best supportive care considered unfit for chemotherapy because of poor performance status and/or multiple medical comorbidities, including renal impairment. The median overall survival (OS) was 3.7 versus 3.6 months for erlotinib versus placebo; (HR=0.94; 95% CI 0.81 to 1.1; p value=0.46).^9^ However, in prespecified subgroup analyses in patients who developed rash after about 28 days (1 cycle), OS improved with erlotinib; (OS HR=0.76; 95% CI 0.63 to 0.92; p value=0.0058 and progression-free survival (PFS) HR=0.66; 95% CI 0.54 to 0.80; p value<0.001); median OS of 2.9 versus 6.2 months.

About 178/302 (60%) of patients in the TOPICAL trial developed rash with erlotinib in the first treatment cycle and >70% developed rash at any time, consistent with what has been reported elsewhere.^9^ In the UK alone, about 30–40% of patients with NSCLC are unfit for chemotherapy,^9^ therefore, assuming a worldwide advanced NSCLC incidence of approximately 1 million,^2^ the potential economic impact of erlotinib use in the 60% of treated patients who develop rash is likely to be significant. We present the results of a cost-effectiveness analysis of erlotinib versus placebo overall and within a predefined subgroup of patients who develop rash within the first (28 days) of treatment. The rationale for erlotinib treatment benefit in patients who develop rash has been discussed extensively.^9–11^

## Patients and methods

### Patients

The patient population included in this analysis were newly diagnosed stage IIIb–IV (pathologically confirmed) patients with NSCLC who were chemotherapy naïve with no symptomatic brain metastases, and deemed unsuitable for chemotherapy by treating physicians based on the Eastern Cooperative Oncology Group (ECOG) performance status (PS ≥2) and/or multiple medical comorbidities including renal impairment.

Patients were followed up until progression or death. The primary end point was OS; secondary end points were PFS, safety and health-related quality of life (HRQoL). PFS was defined as the time between randomisation and progression or death (whichever occurred first). Progression was based using the response evaluation criteria in solid tumours (RECIST). Prespecified subgroup analyses included whether or not patients developed treatment-related rash within the first 28 days (first-cycle rash), gender and histology. HRQoL using EuroQoL EQ-5D-3L (EQ-5D) was assessed monthly. Randomised patients who took study medication were included in this analysis. Rash was assessed using a subjective 5 point scale graded from: no rash (grade 0), erythema alone (grade A), erythema with papules (grade B), erythema with papules and pustules (grade C) and erythema with papules and confluent pustules (grade D). Rash is a common side effect of erlotinib. In this analysis we included patients with all rash grades, regardless of expected relationship with study treatment. Patients considered to have an outcome of rash were those recorded to have a rash score of A–D. Those with a grade 0 were considered to have no rash.

### Treatments

The main comparison is patients randomised to erlotinib who developed first cycle rash (ER) versus placebo. All patients were allowed to receive immediate or delayed palliative chest radiotherapy and/or radiotherapy to metastatic sites as appropriate.

Patients received erlotinib (150 mg) or matching placebo daily until progression. Dose reductions to 100 mg or 50 mg were allowed. Patients who took erlotinib but did not develop rash (ENR) are discussed in the sensitivity analyses.

### Costs/resources

The costs relevant for this analysis were costs from drug (erlotinib), radiotherapy, additional anticancer treatments (eg, other tyrosine-kinase inhibitor (TKI) or mono chemotherapy), patient management (hospital clinic visits, day cases and hospital admissions) and managing important treatment related-adverse events (AEs; eg, diarrhoea and rash). Resource use was collected monthly on the case report forms (CRF). Unit prices were taken from hospital pharmacy records, published NICE reports^7^ ^8^ ^12^ (where available), published literature,^13^ ^14–16^ National Health Service (NHS) reference costs^17^ and the British National Formulary (BNF, 2012),^18^ without adjusting for inflation. Costs were estimated in UK (£) sterling. No discounting was applied to costs or health benefits <1 year; discounting at 3.5% per annum was applied in the second year; nearly all patients (>99%) progressed/died by 2 years.

#### Drug use

Erlotinib price was set at £54.37/tablet for 150 mg, £44.12 for 100 mg and £25.21 for 50 mg.^7^ ^8^ Drug use was determined from the recorded number of tablets dispensed and returned. Drug cost per patient was estimating by multiplying the duration of erlotinib use by the unit price. For placebo, drug cost was set to zero. Where additional treatments were given (for palliation), unit costs were identified.

#### Supportive care

The mean price of palliative radiotherapy (including planning) for advanced stage patients with NSCLC was assumed to be £120 per visit using NHS reference prices (2011–2012).^17^ The total radiotherapy cost is computed by multiplying the duration of therapy by the unit price. Costs for additional anticancer treatments were included in the analysis; the daily price of gemcitabine/carboplatin was assumed to be £47.50; for vinorelbine it was £19.18.^14^ ^15^

#### Clinic visits/admissions

Resource use (hospital and additional clinic visits, day cases, night hospital stays) were recorded on the CRFs. Only hospitalisations from AEs that were recorded as definitely/probably/possibly treatment related were used to compute costs of hospital overnight stays. The price/day for a clinic visit was £100; day case was £670 and an additional £730 for overnight stay (ie, £1400 including admission).^17^

#### Adverse events

Clinically important grade 3 and above serious adverse events (SAEs) with >5% frequency were: rash, diarrhoea and dyspnoea;^9^ other toxicity rates (≤1%) were similar between treatments and expected cost differences were negligible. Patients with *any* grade (maximum AE grade) were included; it was assumed that palliative treatment was taken, even if AEs were milder. For rash and diarrhoea, the costs per day were set to £4.30 and £8.59, respectively;^16^ the cost of morphine at 15 mL/day is about £0.22; steroid use (dexamethasone) based on £13.80 per 100 tablets with standard doses of 3 tablets of 4–8 mg per day was set at £0.42/daily dose; with salbutamol assuming £0.13/daily dose.^18^

Duration of AEs was computed from their start/end dates; daily unit prices of medications for treating AEs (following UK practice) were computed based on a monthly course.^18^ The total cost (per patient) was computed by adding component costs: drug cost, supportive care, palliative radiotherapy (RT), additional clinic visits, hospital day cases, hospital admissions and treatments for SAEs.

### Utilities

The EQ-5D was used to construct health utilities for economic evaluation. The EQ-5D consists of 5 scales (mobility, self-care, usual activities, pain/discomfort and anxiety/depression) collected from baseline until progression/death. Responses were converted into utilities using a UK social tariff based on the time trade off method.^19^ Missing utility data were handled through multiple imputation (MI) techniques. Responses to EQ-5D were captured on paper CRFs during clinic assessments (monthly in year 1 and 6 monthly thereafter).

### Cost-effectiveness analysis

A patient level (Partitioned Survival) cost utility analysis was undertaken. The OS, PFS and post-progression survival (PPS) were determined for each patient. Mean EQ-5D utilities over time were estimated for pre and postprogression periods and multiplied by corresponding survival times to derive QALYs for each patient. The total costs were then modelled to derive mean costs. Finally, mean incremental costs, QALYs and the incremental cost-effectiveness ratio (ICER) were derived.

### Sensitivity analysis

One way sensitivity analyses were carried out, varying resource use by ±20%. In addition, a probabilistic sensitivity analysis (PSA) was carried out using Monte-Carlo simulation (10 000 simulations) using multivariate methods.^20^

For each data set simulated, the incremental net benefit (INB) was computed using the relationship: INB=λ*Δ_E_–Δ_C_, where λ is the CE threshold, Δ_E_, the mean incremental effect and Δ_C_ the mean incremental cost. The proportion of INBs above or below varying values of λ (ranging from £1000 to £100 000) was used to approximate the probability of cost-effectiveness.

### Statistical analysis

A patient level statistical modelling approach was used to determine mean incremental costs and effects. Mean OS and PFS were determined using Kaplan-Meier methods. Utilities were modelled using linear mixed effects models for repeated measures adjusting for baseline and for whether the observed utility occurred preprogression or postprogression. Mean incremental QALYs were subsequently derived. No extrapolation of OS and PFS was carried out (>99% of patients had died at the time of analysis).

Total costs were modelled using a generalised linear model assuming a gamma distribution to derive the incremental mean costs.^21^ Since costs are positive (>0) and skewed, a gamma distribution was considered to be adequate,^21^ ^22^ although this requires a small increment of 0.001 to be added if costs are zero for modelling purposes. MI methods with a maximum of three data sets were used for missing data. The resulting SEs from MI were used to revise estimates of the ICER in sensitivity analyses. Simulation of cost and effects for PSA was carried out using multivariate methods^23^ by generating data from a Normal Copula.^24^ All analyses were conducted using SAS V.9.3.

## Results

### Baseline characteristics

**Figure 1:**
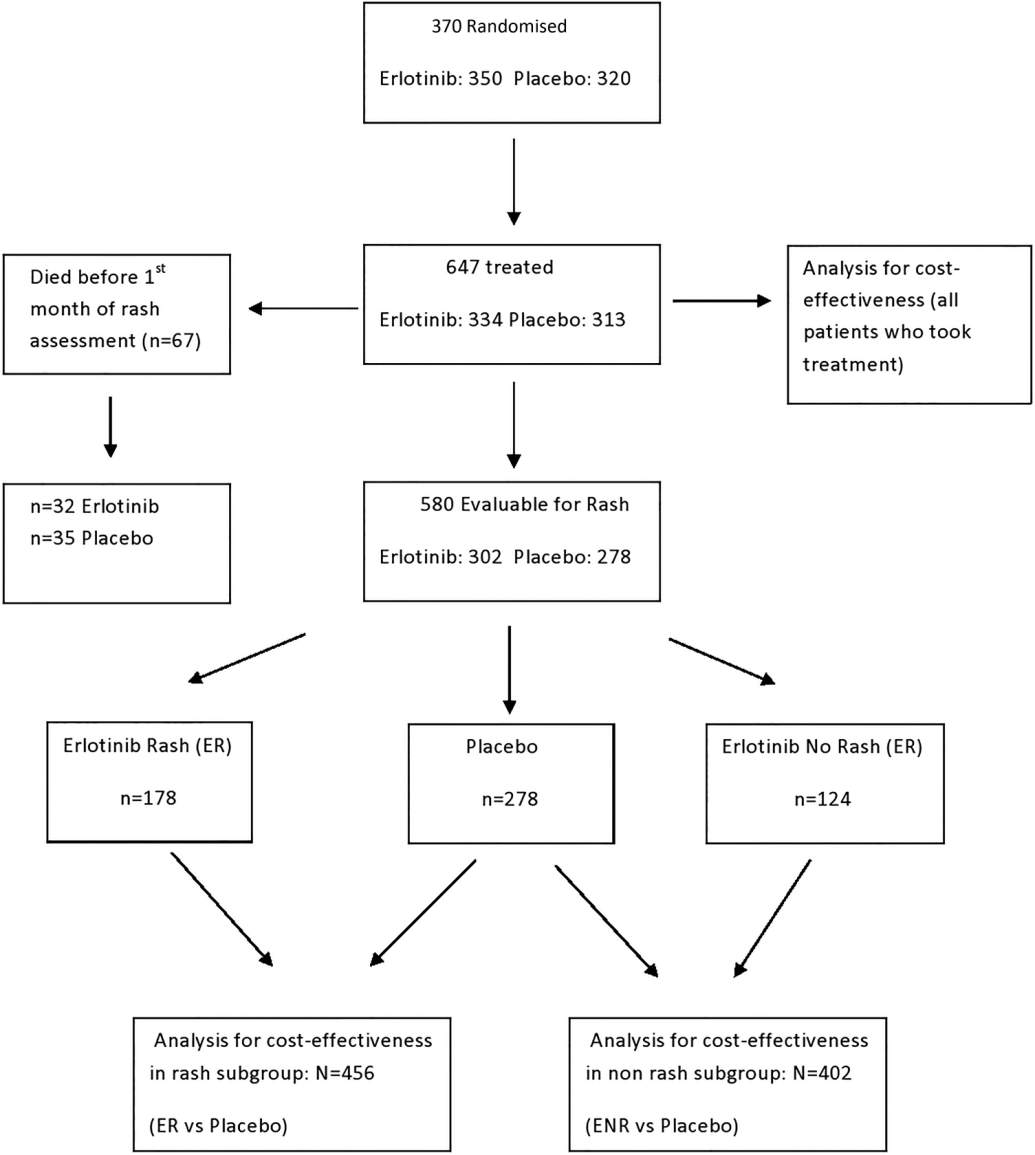
Consort diagram.

Between 2005 and 2009, a total of 670 (350 erlotinib; 320 placebo) patients were randomised across 78 centres in the UK. From 334 versus 313 patients who took study treatment, a prespecified subgroup of 302 erlotinib versus 278 placebo was evaluable for first cycle rash (figure 1 CONSORT). The main comparison of interest in this CE analysis is the subgroup ER (n=178) versus placebo (n=278), since overall there were no differences between erlotinib and placebo; 178/302 (59%) developed rash in the first cycle (ER group) and 124/302 (41%) took erlotinib and did not develop rash in the first cycle (Erlotinib non-rash (ENR) group); 5/313 (2%) on placebo had rash. Patients with ENR were included in a sensitivity analysis. Baseline characteristics were generally similar between groups (table 1). Although there appeared to be a difference for smoking status (except never-smokers), a multivariate analysis^9^ showed that overall survival was similar between ex-smokers and never-smokers (HR 0.98). Also, the efficacy of erlotinib may be reduced in patients who currently smoke, but the absolute difference of 24% versus 37% (p=0.003) does not materially impact the estimate of the QALY and ICER; and, furthermore, we have shown that efficacy is improved in the erlotinib-rash group.

**Table 1:** Summary of baseline characteristics*

	Overall			Rash subgroup		
	Erlotinib (N=350)	Placebo (N=320)	p Value	Erlotinib (N=178)	Placebo (N=278)	p Value
Age						
Median	77	78	0.899	78	78	0.991
Range	(45–91)	(51–91)		(51–91)	(45–91)	
Gender						
Female	135 (39%)	126 (39%)	0.833	69 (39%)	108 (39%)	0.984
Male	215 (61%)	194 (61%)	0.834	109 (61%)	170 (61%)	0.983
ECOG						
0–1	54 (15%)	53 (16%)	0.692	37 (21%)	50 (18%)	0.459
2	194 (55%)	185 (56%)	0.539	103 (58%)	165 (59%)	0.756
3	102 (25%)	90 (27%)	0.771	38 (21%)	63 (23%)	0.741
Cell type						
Adenocarcinoma	133 (38%)	123 (38%)	0.908	63 (35%)	103 (37%)	0.718
Large cell	15 (4%)	15 (5%)	0.801	7 (4%)	15 (5%)	0.477
Squamous	136 (39%)	127 (40%)	0.825	75 (42%)	114 (41%)	0.810
Other NSCLC	66 (19%)	55 (17%)	0.574	33 (19%)	46 (17%)	0.582
Smoking status						
Smoker	124 (35%)	119 (37%)	0.631	43 (24%)	104 (37%)	0.003
Ex-smoker	207 (59%)	183 (57%)	0.608	122 (69%)	158 (57%)	0.012
Never smoked	19 (5%)	18 (6%)	0.911	13 (7%)	16 (6%)	0.509

*Only those patients who took study drug were included in the analysis.

ECOG, Eastern Cooperative Oncology Group; NSCLC, non-small cell lung cancer.

### Costs/resources

Erlotinib was taken as 150 mg tablets by about 83% of patients, without any dose reductions; 15% and 2% of patients reduced dose to 100 mg and 50 mg, respectively. In the rash subgroup this was 79% for 150 mg tablets, 20% for 100 mg and 1% for 50 mg tablets, respectively. Hence, after taking into account dose reductions and dose delays, the mean cost of erlotinib (table 2) was £6863 overall and £7544 in the rash subgroup. For ER versus placebo, additional chemotherapy/TKI costs after progression on the erlotinib arm (n=7) and placebo group (n=5) were £182 versus £270, respectively (mean difference £88, p value=0.852); mean costs for palliative radiotherapy were £302 versus £235 (p value=0.0449); mean duration of radiotherapy was longer with erlotinib (2.5 vs 2.0 days; p value=0.3412), although the proportion of patients who received radiotherapy was similar (table 2 and online supplementary table S1). More patients were hospitalised for treatment-related SAEs on ENR versus placebo: 22/178 (12%) versus 15/278 (5%); mean costs of treatment related SAEs were, therefore, £356 versus £184 and mean total costs were £9949 versus £2058, respectively.

**Table 2:** Model inputs: unit prices for resource use and summary of costs

Costs Item	Estimated unit price (£)	Rash subgroup			Overall (£)		
		Erlotinib (rash) (£)	Placebo/SC (£)	Difference (p value) (£)	Erlotinib	Placebo/SC	Difference
		N=178 Mean (SE)	N=278 Mean (SE)		N=334 Mean (SE)	N=313 Mean (SE)	
Erlotinib*	54.37/tablet	7544 (764)	0	7544	6863 (674)	0	8074
Supportive care:							
Palliative RT†	120/visit	302 (52)	235 (27)	67 (p=0.0449)	350 (48)	242 (39)	108
Additional treatment‡	See note c	182 (65)	270 (99)	−88 (p=0.85)	190 (59)	264 (76)	−74
Patient management:							
Hospital clinic visit§	100/visit	629 (57)	624 (53)	5 (p=0.46)	663 (49)	654 (40)	9
Hospital day case§	670/day case	274 (65)	323 (131)	−49 (p=0.69)	285 (71)	356 (95)	−71
Hospital admission§	730/night	744 (163)	475 (134)	269 (p=0.0352)	775 (149)	534 (122)	241
Adverse events¶	See note e	221 (34)	114 (27)	107 (p<0.001)	264 (40)	181 (32)	83
Total mean cost (SE)** (95% CI)		**9949 (724)** (8530 to 11 368 )	**2058 (185)** (1695 to 2420)	7891 p<0.001)	**9210 (711)**	**2121 (199)**	**7089**
Incremental Cost (SE)**		**7891 (614) (95% CI 6999** to **8783)**			**7089 (589) (95% CI £5935** to **£8243)**		

*Cost as £1631.53 for 30 tablets (150 mg tablet) or depending on dose; +Unit price based on national NHS tariff (NICE report 2011).^7^ ^9^

†Palliative RT: Diagnosis and treatment of lung cancer update (2011).^9^

‡On the Placebo arm, 7 patients took additional chemotherapy (carboplatin/gemcitabine (n=5) erlotinib (n=2)) after progression; on the erlotinib arm, patients took carboplatin (n=2), vinorelbine (n=2), Fragmin (n=1).^11^ ^12^

§Additional clinic visits and day visits irrespective of reason; unit prices taken from NHS reference costs 2010–2011; Hospital nights stayed as a result of treatment-related serious adverse events.^13^

¶Total costs for diarrhoea, rash and dyspnoea; duration of each AE was computed from the date of onset of the event to date resolved. Rash unit price taken from Lewis *et al* 2010^14^; morphine dose of 15 mL/daily is about £0.22/day; steroid use (dexamethasone) based on £13.84 per 100 tablets and taking 3 tablets of 4–8 mg per day gives £0.42/daily dose; inhaler: salbutamol, £0.13/daily dose.^15^ Mean diarrhoea costs were £14 versus £2; mean rash costs were £68 versus £14.90 and mean dyspnoea costs were £139 versus £97.

**Determined using a generalised linear mixed model assuming gamma distributed costs for Erlotinib+Rash versus Placebo.NHS, National Health Service; NICE, National Institute for Health and Care Excellence; RT, radiotherapy.

### Efficacy

The mean and SE for OS was 7.08 (0.48) versus 6.41 (0.44) months. For PFS, this was 4.95 (0.36) versus 3.80 (0.29) months, respectively (erlotinib vs placebo). In the rash subgroup, OS was 9.08 (0.65) versus 6.91 (0.43) months (table 2); and PFS was 6.22 (0.51) versus 4.19 (0.32) months for ER and placebo, respectively.

### Utilities and QALYs

About 98% of EQ-5D forms were completed at baseline; patients alive at 1 year, 32/40 (80%) versus 34/43 (79%) had complete EQ-5D data for ER versus placebo, respectively. Missing data between groups were similar at other time points. As expected, HRQoL was better before progression (table 1): pre-progression utility was 0.6482 (0.009) versus 0.6438 (0.011); for postprogression, mean (SE) utilities were 0.5517 (0.016) versus 0.5760 (0.0140) for erlotinib versus placebo, respectively. In the rash subgroup, EQ-5D utility improvement was higher prior to disease progression: 0.6407 (0.017) versus 0.6193 (0.015); mean difference was 0.0214 (95% CI, -0.0122, 0.0651; p value=0.3408); for post-progression, mean (SE) was 0.5548 (0.0255) versus 0.5756 (0.0200); mean difference of -0.0243 (95% CI, −0.0084, 0.0429; p value=0.5229) for ER versus placebo, respectively.

The mean QALY was 0.365 versus 0.3303 overall, yielding an incremental QALY of 0.035. In the rash subgroup, the mean QALY was, respectively, 0.467 versus 0.337 for ER versus placebo, yielding a statistically significant mean incremental QALY of 0.139 (95% CI 0.0341 to 0.2359; p value=0.0070), in favour of erlotinib. The improved QALY within the rash subgroup appears to be due to improved survival (table 3), notably PFS. Hence, the mean incremental cost was £7090 and an overall ICER of £202 571/QALY.

**Table 3:** Model inputs: effectiveness measures

	Overall		Rash subgroup	
	Erlotinib (N=334)	Placebo (N=313)	Erlotinib (N=178)	Placebo (N=278)
Mean OS (months)	7.08 (0.48)	6.41 (0.44)	9.08 (0.65)	6.91 (0.43)
Mean PFS (months)	4.95 (0.36)	3.80 (0.29)	6.22 (0.51)	4.19 (0.32)
Mean PPS (months)	2.13 (0.250)	2.61 (0.236)	2.86 (0.41)	2.72 (0.27)
HR (OS)	0.92		0.76	
(95% CI; p value)	(0.79 to 1.08; p value=0.32)		(0.63 to 0.92; p value=0.005)	
HR (PFS)	0.81		0.66	
(95% CI; p value)	(0.70 to 0.95; p value=0.0102)		(0.54 to 0.80; p value<0.0001)	
Utilities				
Preprogression EQ-5D (mean, SE)	0.6482 (0.009)	0.6438 (0.011)	0.6407 (0.017)	0.6193 (0.015)
Postprogression EQ-5D (mean, SE)	0.5517 (0.016)	0.5760 (0.014)	0.5548 (0.0255)	0.5756 (0.020)
QALY (years)*	0.365 (0.0272)	0.3303 (0.0245)	0.487 (0.0432)	0.3472 (0.0260)†
Incremental QALY (mean SE)‡		**0.035 (0.0163)**		**0.139 (0.0113)**

*This is computed as (preprogression utility)×PFS+(postprogression utility)×PPS.

†Statistically different between erlotinib and placebo (p value: 0.0070).

‡Erlotinib versus placebo.

PFS, progression-free survival; PPS, postprogression survival; OS, overall survival.

In the rash subgroup, the mean incremental cost was £7891 (95% CI £6999–£8783), but with better HRQoL/utility resulting with a base case ICER of £56 770/QALY (table 4). The incremental cost excluding erlotinib cost was £347 and the ICER was £2496/QALY in the rash subgroup. The mean incremental net benefit (INB) for erlotinib is not realised until one is prepared to pay in excess of about £202 571 overall and £56 770 in the rash subgroup.

**Table 4:** One way sensitivity analysis (rash subgroup)

Parameter	Variation (%)	ICER (£)
Base case		**56 768**
Erlotinib cost	−20	45 821
	+20	67 530
Radiotherapy costs	−20	56 823
	+20	55 953
Hospital admission costs	−20	54 018
	+20	58 758
Preprogression utility	−20	97 671
	+20	48 945
Postprogression utility	−20	160 019
	+20	49 845
Missing data adjustments*	**–**	<58 400

*Using multiple imputation.

### Assessing uncertainty

Sensitivity analyses were conducted for the rash subgroup only. However, as part of the sensitivity analyses, the impact on the ICER was observed from patients in the ENR group who took erlotinib—since these patients would contribute towards the costs of erlotinib in practice,for at least one cycle.

**Figure 2:**
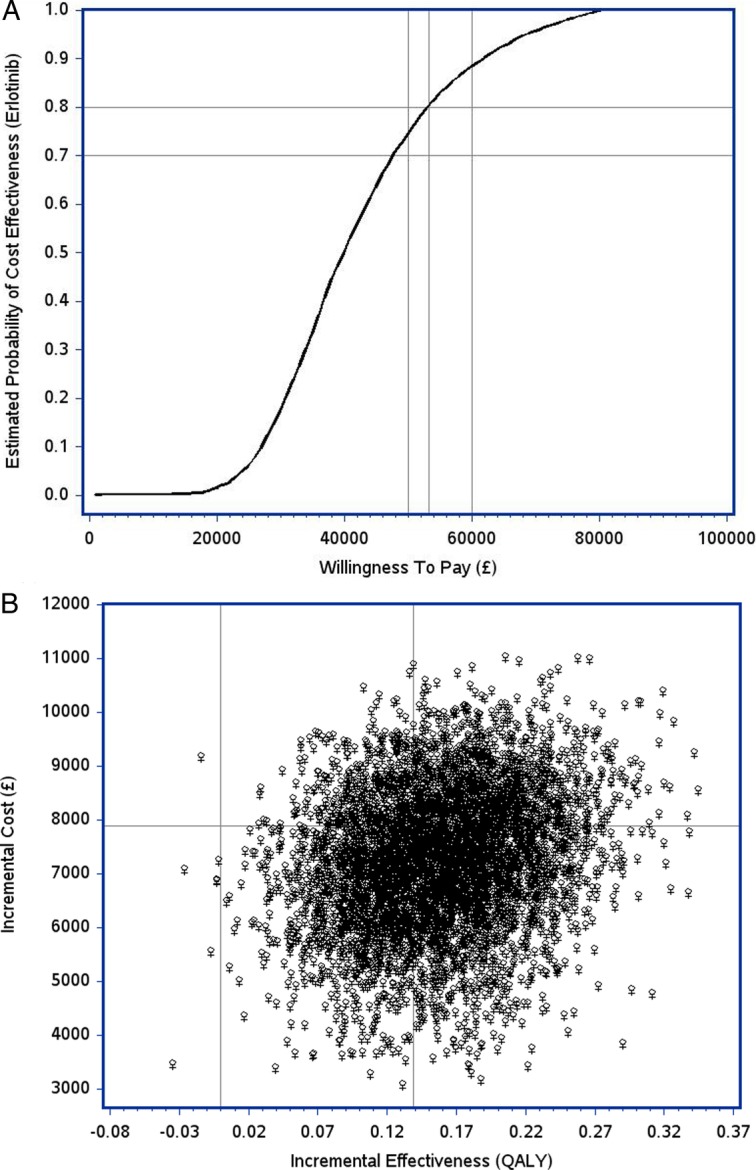
Cost-effectiveness results. (A) *Cost-Effectiveness Acceptability Curve (CEAC): ER* versus *Placebo/SC for rash subgroup.* Note: Vertical reference lines are CE threshold values of £50 000 and the observed cost/QALY (£56 770). The horizontal reference line is 0.8. (B) *Cost-Effectiveness Plane: ER* versus *Placebo/SC (rash subgroup). Note*: The first vertical reference line is 0. The horizontal and second vertical reference lines are observed incremental effect (0.139) and observed incremental cost (£7891), respectively.

Results from one way sensitivity analyses are shown in table 4. The ICER was most sensitive to changes (±20%) in erlotinib costs, and utilities ranged from £45 821/QALY to £67 530/QALY. Most ICERs remained within 5% of the base case after ±20% adjustments. The mean ICER from PSA was £57 120 with 5% and 95% quantiles ranging from £29 438 to £89 550. The estimated probability of cost-effectiveness of erlotinib at CE thresholds between £50 000 to £60 000 was 80% (figure 2A). The CE plane (figure 2B) shows costs and benefits scattered in the north east quadrant where incremental effects are generally positive (erlotinib better), but with higher costs.

Patients who started erlotinib but did not develop rash after the first cycle (ENR) are considered to contribute towards drug costs during the first cycle. Patients with ENR who took erlotinib for at least one cycle had worse outcomes: OS and PFS HRs of 1.30 and 1.09 compared to placebo, with mean (SE) OS and PFS of 5.7 (0.69) and 4.2 (0.55) months, respectively.^9^ These patients are unlikely to continue erlotinib if rash is not observed within the first cycle (but may continue to receive SC). The mean cost of 28 days of erlotinib after accounting for dose delays and reductions was £1279. The total mean erlotinib cost was, therefore, £8187. Hence, after adjusting for all other costs (AEs, supportive care including RT, hospital visits) during the PFS period, the incremental cost and ICER increased to £9578 and £68 906, respectively, assuming an incremental QALY of 0.139.

The base case ICER was robust to differences in the models and methods of handling missing utility data. When MI was used, the ICER did not increase (table 4) beyond £58 400 (range £55 452 to £58 400). The SEs of incremental costs and QALYs increased slightly from £614 to £689 and 0.0113 to 0.0145, respectively, after taking into account missing data, using MI. A summary of ICER results are shown in table 5.

**Table 5:** Summary of results from Cost Utility Analysis (CUA)

Scenario	Incremental costs	Incremental effects	ICER (5th, 95th centile)
Overall (base case)†	£7090	0.139	£202 571
Rash subgroup			
Base case‡	**£7891**	**0.139**	**£56 770** (£29 438 –£89 550 )
Excluding erlotinib costs	£347	0.139	£2496 (£1120–£3895)
Including ENR 1st cycle drug costs§	£9578	0.139	£68 906 (£44 165–£93 276 )

†Erlotinib versus placebo (n=647).

‡ER versus placebo (n=456).

§ER (including ENR first cycle erlotinib costs) versus placebo.

ENR, Erlotinib non-rash; ICER, incremental cost-effectiveness ratio.

## Discussion

In this trial in a population of patients with advanced NSCLC considered unfit for first-line chemotherapy, erlotinib did not show cost-effectiveness overall, however, it has potential for being cost-effective in patients who develop first cycle rash. At the time of our original report,^9^ previous phase III NSCLC clinical trials suggested a relationship between rash development and improved survival with TKI treatment.^10^ ^25^ ^26^ This has since been confirmed in a meta-analysis of 33 trials (6798 patients) of NSCLC, from which the authors concluded that skin rash after EGFR-TKI treatment is an effective surrogate marker for predicting clinical outcomes.^11^ Our findings are also consistent with several other studies to report the relationship between TKIs/EGFR antibodies and rash in different cancer types: Bonner, 2010 (head and neck cancer),^27^ Cunningham (2004) and Bokemeyer (2009)^28^ in colorectal cancer.^29^

The monthly cost of erlotinib treatment was £1650^9^ and at least £9112^15^ for six cycles. This trial demonstrated effects in our patients for whom few treatment options are available apart from palliative radiotherapy. OS improved by a median of >3 months^9^; Improvements in HRQoL were also demonstrated with the EORTC-QLQC-30.^9^ Consequently, ER patients show a high probability (about 80%) of being cost-effective at thresholds between £50 000 and £60 000; this threshold is advocated by supplementary NICE guidelines (in the UK) for end of life treatments.^30^ Moreover, at lower CE thresholds (eg, £30 000–£40 000), between 15% to 40% (figure 2A) of our patients with NSCLC unfit for chemotherapy would have a cost-effective clinical benefit (ie, >3 months improvement in OS and improved HRQoL). With few treatment options available for these patients, this is an important finding.

From published trials, the cost/QALY of erlotinib ranges from £18 170 to £89 377 (incremental QALYs ranging from 0.175 to 1.4).^16^ ^31–38^ The observed QALY of 0.139 in ER patients was within this range. Reported ICERs in EGFR mutation +ve patients for other TKI therapies include: afatinib (ICERs between $45 000 (£23 000) to $75 000 (£38 600)/QALY when compared with gefitinib, erlotinib and cisplatin/gemcitabine)^39^ for gefitinib £21 000 (vs gemcitiabine) to £154 000/QALY (vs cisplatin/gemicitabine).^40^ However, these were in trials where patients were fit for standard chemotherapy. Non-TKI's such as pemetrexed reported ICERs between £18 672 (vs BSC)^41^ to £49 000/QALY (vs BSC).^42^ Crizotinib: ICERs ranging from about £41 500/QALY^43^ versus docetaxel to $216 000/QALY when compared with pemetrexed.^44^

In a recent review of licenced treatments for NSCLC, it was highlighted that the elderly frail population were unrepresented in the majority of lung cancer trials and there remains considerable uncertainty in assessing the cost-effectiveness of treatments for this group of patients.^45^ Moreover, many of these trials did not report the cost/QALY, which would be helpful to decisionmakers. Interestingly, in a phase II erlotinib trial that did include elderly patients (fit for chemotherapy), the reported ICER was €395 400 (£341 198/QALY).^46^

In the UK, the NICE supplementary guidance recognises that during end of life, the standard CE thresholds of £20 000–£30 000 per QALY gain may be inadequate.^31^ ^47^ Two of these three criteria (short life expectancy and ≥3 months improvement in survival) were satisfied. The third criteria: ‘treatment is licensed or otherwise indicated for small patient populations’ is not clearly satisfied; poor performance patients with NSCLC is not considered to be a ‘small’ patient population, despite this population being understudied.^45^ A ‘small population’ should not *normally* ‘exceed 7000 new patients per year’ or should be a ‘small group within larger populations’.^31^

There are several strengths in our analyses. First, the TOPICAL trial was designed to prospectively record health economic data (eg, collecting resource use and utility data). Second, >99% of patients died, therefore, the uncertainty in modelling future health benefits is largely absent. Third, utility data were collected until progression/death for all patients, thereby providing reliable mean postprogression utility estimates. Moreover, the EQ-5D utility data had good completion rates. Despite short survival times, costs and benefits were adequately captured.

A strength of this research is the low rate of EGFR mutation-positive tumours in our population. Currently, NICE recommended EGFR mutation-positive locally advanced or metastatic NSCLCs are treated with TKIs (gefitinib, erlotinib or afatinib) regardless of their performance status. It is possible that if ER patients were to be compared with a similar TKI, the ICER may be higher due to smaller differences between groups. Several other CE analyses exist that compared TKIs against placebo and were approved by NICE.^31^ It is also acknowledged that the analysis associated with rash was not based on a randomised comparison, so there may be some unknown confounders. We excluded deaths prior to the first cycle, but there were only 32 (9%) versus 35 (11%), which was not statistically significant, so unlikely to impact the results (because the survival time was <1 month in both treatment groups, the mean OS and PFS would be very similar). In addition, despite being statistically significant differences of 37% versus 24% in smoking status (p=0.003) with median OS improvement of 2 months (erlotinib vs placebo) for ex-smokers and 1.4 months for current smokers, this did not translate to a meaningful QALY difference. The mean QALY was 0.11 and 0.15 (p=0.1580) for current and ex-smokers, respectively. This suggests that the observed differences in smoking status did not appear to result in meaningful differences in the ICER: about £47 000 (ex-smoker) to £64 000 (current smoker) assuming the same incremental costs. These values of the ICER are within the range of the sensitivity analyses for the overall ICER.

The low EGFR mutation rate (5%) was insufficient to establish the cost-effectiveness of erlotinib for poor performance patients whose tumours are EGFR mutation +ve. In any case, the target population in this trial may not require the (expense of) testing for EGFR mutation status and patients could stop taking erlotinib after the first cycle of rash. In this trial 124/302 (40%) patients without rash continued to receive erlotinib. With the proposed treatment strategy, 40% of patients who took erlotinib would fall into this category and reduce the costs of erlotinib. Therefore, the cost saving could be much higher. Interestingly, all patients who tested positive for EGFR mutation developed rash.

Using the findings from our trial, we estimate that of 100 patients needing to be treated with erlotinib, 60 develop treatment-related rash, who are likely to benefit. The inclusion of the ENR costs for the first cycle (28 days) is justified because it represents a health resource consumed, although these patients are unlikely to improve. The ICER was £56 770/QALY excluding the cost of treating the 40% of non-rash patients for one cycle, and £68 906/QALY including this cost. Both are within the range (£30 000 to £154 000/QALY) of other estimates for gefitinib and afatinib as first-line therapy for EGFR-mutation-positive patients with NSCLC. Although the mean cost from 28 days of erlotinib use in patients with ENR has been included (when calculating the ICER of £68 906), the ICER does not reflect the negative effects and poorer QoL (dis-utility) from erlotinib use in the patients with ENR group after first cycle of treatment. Therefore, the higher ICER of £68 906, while reflecting total erlotinib usage, may not reflect the efficiency of erlotinib for the target population.

In conclusion, erlotinib offers a potentially cost-effective treatment option for the subgroup of predominantly poor performance patients with NSCLC with EGFR wild-type tumours who develop first-cycle rash and who are considered unfit by clinicians to be treated with first-line chemotherapy.
