# Correction

**DOI:** 10.1136/bmjopen-2014-006733corr1

**Published:** 2017-04-07

Khan I, Morris S, Hackshaw A, *et al*. Cost-effectiveness of first-line erlotinib in patients with advanced non-small-cell lung cancer unsuitable for chemotherapy. *BMJ Open* 2015;**5**:e006733. doi: 10.1136/bmjopen-2014-006733

In figure 1, the top square currently reads ‘370 Randomised’. This should read ‘670 Randomised’ as mentioned earlier in the text.

